# Smoothing out the transition of care between maternity and child and family health services: perspectives of child and family health nurses and midwives’

**DOI:** 10.1186/1471-2393-14-151

**Published:** 2014-04-27

**Authors:** Kim Psaila, Sue Kruske, Cathrine Fowler, Caroline Homer, Virginia Schmied

**Affiliations:** 1 School of Nursing and Midwifery, University of Western Sydney, Sydney, Australia; 2 Director Queensland Centre for Mothers & Babies, The University of Queensland, Brisbane, Queensland, Australia; 3 Tresillian Chair for Child & Family Health, Centre for Midwifery, Child & Family Health, Faculty of Nursing, Midwifery & Health, University of Technology Sydney, Australia; 4 Faculty of Nursing, Midwifery & Health, University of Technology Sydney, Sydney, Australia; 5 School of Nursing and Midwifery & the Family and Community Health Research Group, University of Western Sydney, Sydney, Australia

**Keywords:** Transition of care, Maternity services, Child health services, Midwives, Child and family health nurses, Communication, Collaboration, Continuity

## Abstract

**Background:**

In Australia, women who give birth are transitioned from maternity services to child and health services once their baby is born. This horizontal integration of services is known as Transition of Care (ToC). Little is known of the scope and processes of ToC for new mothers and the most effective way to provide continuity of services. The aim of this paper is to explore and describe the ToC between maternity services to CFH services from the perspective of Australian midwives and child and family health (CFH) nurses.

**Method:**

This paper reports findings from phase two of a three phase mixed methods study investigating the feasibility of implementing a national approach to CFH services in Australia (the CHoRUS study). Data were collected through a national survey of midwives (n = 655) and CFH nurses (n = 1098). Issues specifically related to ToC between maternity services and CFH services were examined using descriptive statistics and content analysis of qualitative responses.

**Results:**

Respondents described the ToC between maternity services and CFH services as problematic. Key problems identified included communication between professionals and services and transfer of client information. Issues related to staff shortages, early maternity discharge, limited interface between private and public health systems and tension around role boundaries were also reported. Midwives and CFH nurses emphasised that these issues were more difficult for families with identified social and emotional health concerns. Strategies identified by respondents to improve ToC included improving electronic transfer of information, regular meetings between maternity and CFH services, and establishment of liaison roles.

**Conclusion:**

Significant problems exist around the ToC for all families but particularly for families with identified risks. Improved ToC will require substantial changes in information transfer processes and in the professional relationships which currently exist between maternity and CFH services.

## Background

Continuity in healthcare has been described as the degree to which a series of discrete healthcare events are experienced as coherent, connected and consistent with the patient’s needs over time [1]. In Australia, universal maternity and child and family health (CFH) services are provided to women and their families via a schedule of universal health contacts within a system of primary health care [2].

In Australia, publicly-funded maternity services offer care during pregnancy, labour and birth and the early postnatal period. Of the women who gave birth in Australian hospitals in 2010, approximately two thirds gave birth in public hospitals, one third gave birth in a private hospital and 1% were born at home or outside the hospital system [3]. Postpartum care in the first week is most commonly provided by midwives, in hospital and in the community through home visits. Most women are discharged from maternity services between two and ten days with a small proportion of women having continuing access to midwives for up to six weeks in some models of care [4,5].

Following discharge from maternity services, women and babies ‘transition’ to ongoing publicly funded CFH services. CFH nurses offer care for children and families at regular contact points from the early postnatal period until commencement of school [6,7]. Health activities and primary prevention screening which are conducted at each contact are governed by a schedule specific to each State and Territory. In 2013 a National Framework for Universal CFH Services [2] was released but this does not include minimum number or age-related universal health contacts. Almost all jurisdictions (except one) recommend at least five contacts, at least half of which are offered within six to eight months of birth [8]. The initial contact occurs within 4 weeks of birth although most jurisdictions recommend this occurs within one to two weeks of discharge from hospital.

The CFH service includes, but is not limited to, developmental surveillance and health monitoring, promotion of parental social and emotional wellbeing, risk identification and health promotion [9,10]. General practice (general practitioners and practice nurses) also provide well child services opportunistically including developmental and preventative health care and immunisations [11]. These services attract an out-of-pocket fee with some subsidy through the public health insurance system called Medicare.

One of the key elements in providing effective continuity of care to women and their families following birth of a baby is a smooth ‘Transition of Care’ (ToC) from one service to another [12,13] such as from maternity services to CFH nursing services. In this context ToC refers to the horizontal integration of services on the same level across the care continuum [14]. The most common point of formal transition for childbearing women and their infants occurs in the postpartum period, from maternity to CFH nursing services and/or to general practice.

The postnatal period is a particularly stressful time for new mothers faced with caring for a baby while simultaneously trying to recuperate after the birth [15,16]. Factors that may contribute to the early postnatal period being particularly stressful for women include adverse physical and emotional health symptoms such as extreme fatigue, genitourinary symptoms, depressed mood and anxiety disorders [17-19]. Some women face additional challenges due to existing prenatal risk factors such as anxiety or stress disorders, low socioeconomic status, domestic violence, substance abuse [19,20]. Maternal stress interferes with maternal infant interaction and has the potential to impact on a child’s health and development [21,22].

The new mother’s experience of stress will vary dependent on the level of functional support (information, practical and emotional support) provided by the her social network of informal (friends, family, peer) and formal (health professionals) structural supports [23,24]. The value of formal structural support from health professionals has been reported in the literature. For example, MacArthur et al [25] found midwife-led, flexible postnatal care, tailored to needs of the women, could help to improve women’s mental health and reduce probable depression at 4 months’ postpartum. While Leahy-Warren et al [23] found that the best predictors of postnatal depression at 12 weeks postpartum were the formal (health professionals) structural support and emotional functional support at birth.

Support in the early postnatal period from midwives [26,27] and from CFH nurses [28] has demonstrated positive outcomes for women including a reduction in postnatal depression. A recent study in Denmark, found that in order to feel secure, families needed to know that they could rely on follow-up support after discharge [29] and having the telephone number of someone to contact if they were worried in the early weeks was a predictor of satisfaction in postnatal services in a large survey of over 5000 women in Queensland [17].

While there is published literature regarding the concept of “transition” within acute based health services [30,31], there is limited information on ToC between midwifery and community based CFH services. In a Swedish study of continuity between maternity and CFH services, Barimani and Hylander [13] found a service gap resulting from a lack of cooperation between midwives and child health nurses. Likewise an Australian study aimed at improving understanding of effective strategies to transfer women from maternity to CFH nursing services in NSW found that there was a lack of formal processes to ensure effective communication and promote collaboration between maternity services and CFH nursing services resulting in fragmentation of care [12]. Despite recognition of the benefits of postnatal support, discontinuity along the maternity and CFH continuum of care appears to be most prevalent at the point of transition from maternity services to CFH services. In order to optimise the transition to motherhood it is essential to explore strategies which would improve continuity of care for women and their families in the early postnatal period.

The Child Health: Researching Universal Service (CHoRUS) study is a three phased mixed methods study investigating the feasibility of implementing a national approach to the provision of universal health services for children and families. In phase one of the CHoRUS study, consultations were held with representatives of the professional associations: Australian Association of Maternal, Child and Family Health Nurses (formerly AAMCFHN now MCaFHNA), Australian College of Midwives (ACM) and key stakeholders such as policy makers and other service providers (e.g. allied health) to determine the facilitators and barriers to the delivery of universal CFH services. One of the key findings in phase one was the difficulties in the ToC of women and their babies from maternity to CFH nursing services [32]. To examine the impact of this and other concerns identified in phase one, we conducted a national survey of midwives and CFH nurses (modified for the two professional groups) in phase two of the CHoRUS study. Items to inform understandings on the method, timing, content and quality of information transferred between services as well as the facilitators and barriers to the ToC of families between hospital-based maternity and community-based child health services were included. The aim of this paper is to explore and describe the process of ToC between maternity services and the CFH service from the perspective of Australian midwives and CFH nurses.

## Methods

### Research design

National surveys of midwives and CFH nurses were conducted. The University of Western Sydney (UWS) Human Research Ethics Committee gave ethics approval for the study.

### Data collection

The national surveys were designed to further explore the issues that were identified by professionals in phase one of the CHoRUS Study and to describe in greater detail the role of midwives and CFH nurses in delivering universal health services to women, their children and families with a larger sample of professionals. Both surveys were developed by the CHoRUS multidisciplinary research team specifically for this study, although some questions were based on a survey of health visitors in the UK [33]. Items were developed from criteria identified by professionals in phase one as being essential for effective transition of care as well as from the literature on ToC [12,34] and continuity [35,36] and from current policy documents [10]. Each item was reviewed for validity by content experts from each of the professional groups including stakeholders from the participating professional associations, (CFH nurses, Midwives, GPs, policy makers and consumers) and members of the research team. Both surveys were piloted using an external convenience sample from both of the professional groups. At each point, the survey was reviewed to assess the relevance of the items and suggested revisions to wording, sequencing, and response alternatives of some items.

This paper reports on 25 items – five related to demographic data and 16 items related to ToC (midwives survey -14 items, CFH nurses survey - 7 items, 5 items were the same in each survey). Several items were followed by text fields requesting clarification of item choice. Specific items on ToC in each survey included: timing of contact with other services, the process of information transfer between services, who completes documentation and when, the information is provided to other services etc. Professionals were also asked to rate their satisfaction with specific aspects of the ToC process e.g. comprehensiveness of information transferred.

Surveys were launched at each professional group’s national conference. In addition, information about the surveys was distributed via professional associations with potential respondents being directed via a web link to an electronic version of the survey on a dedicated CHoRUS study page on the study webpage. Alternatively respondents were able to complete a hard copy version which they then returned by mail to the university. The surveys were available, from May to October, 2011 (CFH nurses) and from October 2011 to February 2012 (midwives). A total of 1098 CFH nurses and 655 midwives responded to the survey. A greater proportion of surveys were completed online (CFH nurses 68%, midwives 77%).

### Data analysis

All data, including data recorded via hardcopy survey, were entered using the Qualtrics online survey platform. Data were exported to MS Excel for cleaning and then transferred to SPSS (21) for further analysis. Descriptive and inferential statistics were used to analyse responses to survey items. Content analysis was used to analyse textual data [37]. A coding list was developed and text responses were then coded into the respective code using the QSR NVivo 9.2 data management program. If the respondent’s meaning was not readily determined it was coded to ‘meaning unclear’.

## Results

### Characteristics of respondents

Phase two surveys were returned by 1098 CFH nurses and 655 midwives. However the number of responses to individual questions varies due to a direction given to respondents to answer questions based on their current role. The majority of CFH nurses and midwife respondents were female with only six CFH nurses and eight midwife respondents being male. The median age of respondents in both groups was 50 years. Across all states and territories, 84.2% of midwives and 82.8% of CFH nurses worked in urban areas with very few working in remote areas (2.9% of midwives and 1.9% of CFH nurses). CFH specialty qualifications were held by 96.7% of CFH nurses and 22.4% of midwives (Table 1).

**Table 1:** Demographic characteristics of respondents

**CFH nurses**			**Midwives**		
Mean age of respondents (years)	51.2		Mean age of respondents (years)	48.3	
Years working as a CFHN	N	%	Years working as a midwife	N	%
Less than 5	203	19	Less than 5	88	14
5 – 10	243	22	5 – 10	88	14
10 – 20	358	33	10 – 20	154	24
More than 20	289	26	More than 20	320	49
N of responses = 100%*	*1093*		N of responses = 100%*	*650*	
Type of employment	N	%	Type of employment	N	%
Full-time	434	40	Full-time	305	47
Part-time	621	57	Part-time	297	46
Casual	43	3.9	Casual	48	7.6
N of responses = 100%	1098		N of responses = 100%	650	
**Qualification**	**CFHN**		**Qualification**	**Midwife**	
	**N**			**N**	**%**
Post-registration certificate	90	9.6	Hospital certificate	210	49
Diploma or degree	140	15	Bachelor of Nursing and post graduate diploma or Masters in midwifery	135	32
Postgraduate certificate or diploma	525	56	Bachelor of Midwifery	23	5.4
Masters’ degree or higher	165	18	Bachelor of Nursing and Bachelor of Midwifery	22	5.1
Other	30	3.3	Other	39	9.1
N of responses = 100%*	*950*			*429*	
**Role**	**CFHN**		**Role**	**Midwife**	
	**N**	**%**		**N**	**%**
CFHN – universal service	646	59	Hospital-based midwife	309	47
CFHN – secondary service	142	13	Community-based midwife	34	5.2
CFHN – tertiary residential unit	18	1.8	Midwife – clinical consultant	37	5.7
CFHN – combination universal, secondary, tertiary	28	2.7	Midwife in private practice	20	3.2
Nurse practitioner, clinical nurse consultant, clinical or nurse educator or Academic	91	8.4	Midwifery group practice/continuity of care model	70	11
CFHN - manager	102	9.4	Manager	46	7.1
Other	71	6.6	Educator or Academic or Other	134	21
N of responses = 100%*	*1098*		N of responses = 100%*	*650*	

*Sum of responses may not total 100% due to rounding.

This was a clinically experienced cohort with just over one fifth (22.7%) of the midwives and less than half (40.9%) of the CFH nurses being employed for less than 10 years. Less than half of the midwives (45.7%) and CFH nurses (40%) worked full-time. However the proportion of full-time employment varied between jurisdictions, that is, Australian states and territories (Table 1).

### Information transfer method

Midwives were asked about the ToC and referral of women and families following discharge from hospital or maternity services. Referral in the form of discharge summaries to CFH nursing services was most common (77.4%) with almost half of the services also referring to general practice (48.2%).

Midwives reported the most common mechanism for transfer of information from maternity to CFH services was ‘direct fax from maternity service to the CFH centre’ (45.2%), and ‘electronic referrals’ (35.7%). This differed across the country. Almost three-quarters (72.4%) of midwives from Western Australia, reported ‘electronic referrals’ as the most common mechanism for ToC, whereas in Queensland, one third (36%) of midwives reported ‘women just presenting or ringing the CFH nurse’.

CFH nurses reported information from maternity services predominantly transferred via an electronic data system (51%), including mandatory birth notification. Email and fax were also common (29.7%) - either directly to the CFH centre or central intake point. The most notable variance was in Queensland where CFH nurses and midwives both reported a high percentage of ‘women just presenting or ringing the CFH nurse’ (40%).

Midwives were asked how ToC took place for women and babies with additional needs or risk factors. In most jurisdictions, midwives reported using a ‘standard’ written discharge summary process most often. This implied that information about the woman and her infant was detailed on a discharge summary and sent to the CFH nursing service or GP without any contact between the two services. Approximately one quarter of all respondents (25.6%) indicated that someone from the maternity service telephoned the CFH nurse or other services for families requiring additional support. Variations were found in the states of Victoria and Tasmania where midwives 51.6% and 58.8% of midwives respectively reported ToC occurring via telephone contact. This suggests that in these two states a higher proportion of midwives prioritised a person-to-person transfer of information for women and families with risk factors.

### Information transfer

When asked about the timing of information transfer, 88.5% of midwives reported routinely sending discharge summaries. However, only three quarters (74.5%) of midwives who worked in private hospitals reported routine discharge summaries being provided for all women compared with almost all (92.9%) of the midwives working in the public sector. A small proportion of midwives (3.4%) reported providing discharge summaries only for women with risk factors identified (8.5% in the private and 2.2% in the public sector). In contrast, midwives in the private sector were less likely to send discharge summaries for women who were judged not to have risk factors compared with the public sector (14.9% vs 3.6%).

The professional responsible for completing the discharge summary was most often the midwife caring for the woman at discharge from hospital. Over two-thirds of the midwives (70%) reported that discharge summaries were completed within two days of discharge (i.e. including those completed on or before the day of discharge). CFH nurses reported that their service received birth notifications or discharge summaries within five days of discharge (82.7%), and an additional 7.2% indicated this occurred by 10 days. A further 10% of CFH nurses reported that their service received summaries greater than 10 days after discharge from maternity services.

Midwives were asked whether their maternity service offered a postnatal home visiting service and to identify the type and length of service provided. The majority of respondents (88.7%) indicated that their service did provide home visiting (Table 2). In maternity services offering home visiting, 47.4% visited at least three times in the first week (Table 2). Additional client information collected by the midwife during the home visiting period recorded in the client record was not routinely included in the discharge summary.

**Table 2:** Type of maternity home visiting services and frequency of contacts

**Type of home visiting**	**Australia**	
	**N**	**%**
Hospital-based midwife visits as part of postnatal service	350	81.4
Caseload or group practice provides midwifery home visits	242	56.3
Community midwife visits at home	183	42.6
Other	47	10.9
N of respondents*	*430*	
Type of contact	Australia	
	N	%
Face to face contact		
3+ visits in first 7 days	176	47.4
6+ visits in first 7 days	26	7
Mean visits in first 7 days		
	2.9	
Standard deviation	1.63	
Phone contact		
3+ calls in first 7 days	107	28.8
6+ calls in first 7 days	30	8
Mean calls in first 7 days	2.3	
Standard deviation	1.76	
N of respondents*	*371*	

By maternity services providing home visiting in Australia.

* Total respondents are those who indicated that their maternity service provided a home visiting service by midwives. Respondents could give more than one response.

Text comments from CFH nurses in relation to the ToC indicated problems with the transfer of information collected by maternity home visiting services to CFH nursing services

*‘Lack of communication between domiciliary midwifery care and CFH centres. CFH nurses often unable to take phone call due to workload. When call returned – domiciliary midwife not available. Perhaps a better electronic systems of handover.’* (CFH nurse, 786 VIC)

*‘The information is not current at times and does not include [domicillary] information--differing levels of communication to add this info some times.’* (CFH nurse, 271 NSW)

Almost one in five CFH nurses (17.8%) reported that their service made first contact with some women in the antenatal period. However, most CFH nurses (83%) reported that their service first contacted women within two weeks of the baby’s birth. An additional 12% reported that their service first contacted women and families within four weeks (Table 3).

**Table 3:** Usual time of CFH nurse first contact with new clients and frequency of receiving necessary information from maternity services in Australia

**Length of time after birth**	**Australia**	
	**N**	**%**
< 2 weeks	431	82.9
2-4 weeks	64	12.3
4-8 weeks	7	1.4
Other	17	3.3
Unsure	1	0.2
N of responses = 100%*		*520*
Frequency	Australia	
	N	%
All the time	115	17
Frequently	338	49.7
Sometimes	180	26.6
Rarely	36	5.3
Not at all	10	1.5
N of respondents = 100%*		*679*

*N includes respondents who report working in universal services and excludes respondents whose services generally see clients antenatally. Sum of responses may not total 100% due to rounding.

### Quality of information transferred

The quality of information included in the discharge summary is critical as it is used by the CFH service to inform the level of support required and the prioritisation of women with higher levels of need [38]. Early engagement of women and the provision of support and access to support through mothers groups, professional services and other resources in the community is especially important for women with or at risk of mental health issues [39].

CFH nurses were asked to indicate how often they perceived that their service received ‘all the necessary information’ about a woman and her newborn from the maternity service to provide ongoing support. Two-thirds (66.7%) perceived that they received all the necessary information from the maternity service ‘all of the time’ or ‘frequently’ (Table 3). Information was reported as received ‘sometimes’ by 26.6% and ‘rarely’ or ‘not at all’ by 6.8% of CFH nurse respondents (Table 3). Similar ratings were provided by the midwives who were asked to indicate whether they believed the information provided in the discharge summary was sufficient for the CFH professional to plan ongoing care. Midwives used a five-point scale, ranging from 1 (insufficient information) through to 5 (more than sufficient information). Midwives rated the information provided in the discharge summary as ‘sufficient’ (45.7%) and ‘more than sufficient’ (26.6%) (Table 4). No variation across states was noted in either the CFH nurse or midwifery scores regarding adequacy of information transferred.

**Table 4:** Midwives rating of the adequacy of information in discharge summaries, by Australian State and Territory

**Rating**	**NSW**		**Vic**		**Qld**		**SA**		**WA**		**TAS**		**ACT**		**NT**		**Aust**	
	**N**	**%**	**N**	**%**	**N**	**%**	**N**	**%**	**N**	**%**	**N**	**%**	**N**	**%**	**N**	**%**	**N**	**%**
1. Insufficient	18	12	4	4.5	9	12	7	20	3	5.8	1	6.3	0	0	3	16	45	9.7
2	34	22	11	12	9	21	6	17	8	15	5	31	2	10	2	11	84	18
3. Sufficient	65	41	41	46	9	48	14	40	23	46	10	63	9	45	11	58	213	46
4	33	21	24	27	10	13	6	17	14	29	0	0	6	30	3	16	98	21
5. More than sufficient*	6	3.8	9	10	4	5.3	2	5.7	4	7.9	0	0	3	15	0	0	26	5.6
Mean rating	2.8		3.2		2.8		2.6		3.1		2.6		4		2.7		2.9	
Standard deviation	1.1		1		1.1		1.3		0.9		0.7		1		0.9		1	
*N of respondents*	*156*		*89*		*75*		*35*		*52*		*16*		*20*		*19*		*466*	

*The sum of responses to the ratings 1-5 may not total 100% due to rounding.

This was not supported by many of the open text responses where midwives indicated that the information persuaded was inadequate. Respondents described this as being due to: staffing issues (shortages or inexperienced staff filling out the forms) and the design of the discharge summary. One midwife wrote:

*[The] information is not always accurate due to limited postnatal care and time in hospital. It would be so much better to actually be able to handover verbally/face to face information re high risk families to the nurse that will be providing the care: currently too much red tape and admin blocks that are all too time consuming. We have lost what the focus for the referral actually is all about: to provide appropriate follow-up for the families that most need the care, not to meet KPI's [key performance indicators] re universal health home visiting…’* (Midwife 119, NSW)

Specific problems included limited options in official documentation on where to provide individualised information especially on social and emotional problems. In public hospital services, psychosocial assessment was reported to be undertaken routinely by 86.9% of midwives, however only 38.9% reported this information being included in discharge summaries. This discrepancy was even more notable in the private sector with 52.3% reporting routine psychosocial assessment being undertaken while only 15.9% including psychosocial details in discharge summaries (Table 5).

**Table 5:** Results of routine psychosocial assessment included in discharge summary

**Screening undertaken routinely?**	**% of midwives employed in public sector**		**% of midwives employed in private sector**		**% of total midwives (public and private)**	
	N	%	N	%	N	%
Yes	291	86.9	23	52.3	314	82.8
No	39	11.6	21	47.7	60	15.8
Unsure	5	1.5	0	0	5	1.3
N of respondents = 100%*	335	100	44	100	379	100
Psychosocial assessment in discharge summary?						
Yes	130	38.9	7	15.9	137	36.2
Sometimes	101	30.2	20	45.5	121	32
No	81	24.3	9	20.5	90	23.8
Unsure	22	6.6	8	18.2	30	7.9
N of respondents = 100%*	334	100	44	100	378	100

### Effectiveness of transition of care

The overall effectiveness of the ToC process from maternity care to CFH services was assessed using a 5 point scale, ranging from 1 (not effective) through to 5 (extremely effective). Only 36.6% of midwives rated the process for ToC for the majority of women and/or babies as effective (4) or extremely effective (5), despite previously reporting information provided in the discharge summary as ‘sufficient’ or ‘more than sufficient’ (72.3%) (Table 4). The transition process for women and/or babies identified with at risk factors for poor physical or mental health outcomes was only slightly better with 40.4% 4 or above (Table 6).

**Table 6:** Effectiveness of transition from maternity to CFHN services for majority of women and for families at risk, percentages

**Rating**	**Majority of families**	**Women and/or babies at risk**
1. Not effective	7.0	7.2
2.	17.7	14.4
3. Somewhat effective	38.7	37.6
4.	28.6	30.4
5. Extremely effective	8.0	10.4
Mean rating	3.1	3.2
Standard deviation	1.04	1.07
*N of respondents*	*486*	*473*

Midwives were asked to explain their rating of effectiveness by responding to an open-ended question. There were 372 textual responses of which, 113 (30%) provided negative feedback regarding the effectiveness of the ToC process. Negative comments included; insufficient or missing individualised data, doubling up of service provision, lack of feedback to midwives from CFH service, staffing issues, and system issues of time lag, difficulty in contacting CFH nurse¸ being actively prevented from contacting CFH nurses directly if concerned about a family.

There were also comments which indicated tensions regarding the ToC. For example, one midwife indicated that women are often coerced into agreeing to transition to the CFH service and then suggests that neither general practice nor the CFH nursing service is able to cater for family’s needs. This midwife wrote:

‘*It is an expectation that women WILL want the [CFH nurse] service, women are approached [about ToC at] 7.30-8 am, often on day 3 when they are vulnerable, a CFHN/stranger walks into the room, with all this information, women know little about the service and generally agree to anything to get the CFH nurse out of their room, not all women can afford to see GP for follow up care/6 week check/concerns about their baby, OR there is a 6-8 week wait for an appointment. CFH nurse appointments only and wait [the waiting time] is similar. "Transition" is dreadful.’* (Midwive 69, VIC)

In contrast, some midwives were concerned that women would not connect with CFH service if left to arrange their own transition, for example:

‘*most mothers are expected to find their child health clinics themselves’* (Midwife 372, QLD)

‘*most low risk women left to self refer but most risk factors may not be evident until after discharge from maternity service*’ (Midwife 608, QLD)

Some midwives indicated that their role in postnatal care is misunderstood by CFH nurses and others emphasised the misunderstanding of midwife’s role and differences in philosophies of care.

*‘[ToC] happens by default - there is no communication and no acknowledgement, appreciation or understanding of our role as important.’* (Midwife 608, Qld)

*‘Very different information and style of care [between maternity and CFH]. The maternity service provides a strength based support and there are many CFH nurses with very old fashioned ideas imposing them on women.’* (Midwife 40, SA)

CFH nurses were also asked to comment on the effectiveness of ToC. Of the 525 responses, 245 provided negative feedback regarding the ToC process. CFH nurses perceived problems were due to: discharge from SCN and NICUs, early postnatal discharge, transitions from private hospitals and a lack of understanding of the CFH nurses’ role. For example:

‘*Very rare to receive recent information from hospital i.e. history etc. Even after making contact and leaving details with SCN* (Special Care Nursery) *babies are often discharged without notifying CFH nurse.’* (CFH nurse 609, Vic)

*‘With early discharge those women in public hospitals are entitled to one or two Domiciliary Home visits to care for breastfeeding issues etc. However, often only have one visit then a quick phone call "is everything alright?" CFH nurse doesn’t get to see mothers until day 7 - 14 so many disasters can and do occur…’* (CFH nurse 994, Vic)

Problems between the private sector and CFH service included lack of follow-up support for women, non referral to either the CFH nursing service or to a GP, inadequate information transfer (delayed or poor quality). The CFH nurses identified:

*‘Electronic birth notification as per legislation. Verbal or written(fax/email) information is patchy and almost never from Private hospitals. We are told this is because hospital midwives are too busy to regularly give a handover to maternal + child Health Nurses.’* (CFH nurse, 546 VIC)

*‘.*.. *often information is not received from the private hospital until requested by the child health nurse following presentation of the mum to clinic or phone call from mum…’* (CFH nurse706, NT)

‘*major [public] hospitals provide home visiting but private patients miss out and it is expected CFH nurse has to pick up the slack especially if premature and early discharge. Certain private hospitals do not necessarily always have good breastfeeding follow-up.’* (CFH nurse 888, Vic)

CFH nurses also perceived that maternity staff underestimated the value of the CFH nursing service and therefore do not actively promote the service to women. This in turn may contribute to a poor ToC for families. For example:

‘*I do not believe that maternity staff (clerical and nursing/midwifery) understand the nature of the ongoing service that (CFH nurses) provides and hence undervalue the importance of timely handover of information that would aid service delivery*.’ (CFH nurse NSW, 539)

### Improving transition of care

Text responses regarding the effectiveness of the TOC process included a small number of suggested strategies to improve the TOC from midwives (4%) 16/372 and from CFH nurse (6%) 23/525. A specific liaison role to support ToC was the most common suggestion by both groups. Other suggestions included: joint visits to families by CFH nurses and midwives to facilitate handover, all women provided with information antenatally or during hospitalisation on the value of CFH nursing service, an opt out system of consent to ensure all women are contacted by CFH nursing service, improve information content, improve communication pathways for vulnerable families, allocation of CFH nurses to visit hospitals to inform women and staff of the CFH service.

When asked to identify from a list of strategies the one they believed would best improve the ToC, the majority (57%) of midwives recommended implementing an electronic data system for easier sharing of information. A quarter (24.5%) selected the use of the same or similar assessment tools. This was supported by open text responses:

‘*I would prefer an ‘opt out’ system of consent to contact, as currently a lot of work on part of hospital midwife, central administration, local CFH nursing service administration and local CFH nurse just to get a client on our list. Also we miss quite a few who have signed consent but we never get the paperwork.*’ (CFH nurse 881, SA)

‘*Regular visiting to the maternity unit by CFH nurse, weekly case conferencing with midwives, CFH, Indigenous Health Worker, Indigenous Liaison worker, social worker, Child Protection officer and monthly attendance from the Aboriginal medical service. Faxed referrals are received from the private hospital.*’ (CFH nurse 1014, SA)

The majority of midwives (53%) suggested regular meetings between maternity and CFH services to discuss transition and problems experienced by ‘at risk’ families. Approximately one-third (32.4%) suggested verbal handover as a strategy.

## Discussion

The aim of this paper was to explore and describe the ToC between the maternity service to the CFH nursing service from the perspective of Australian midwives and CFH nurses. A smooth ToC process is promoted to facilitate effective continuity of service [40,41]. ‘Continuity of service’ refers to the provision of service consultations and/or interventions which are linked into a coherent care strategy. A previous qualitative study by Homer et al. [12] and published findings from phase one of the CHoRUS study [32] report numerous problems with the ToC from maternity to CFH services including ineffective communication pathways, restricted information transfer, duplication of service provision. In phase two of the CHoRUS study, presented in this paper, we found that ToC for the majority of women and families occurred via an electronic referral or direct fax of maternal obstetric information from the maternity service to the CFH nursing service (93% public vs 74% private hospitals). Participants reported a small proportion of families (17% overall) are requested to contact the CFH nursing services themselves though this was much higher (36%) in Queensland.

The National Safety and Quality Health Service Standards [42] call for ‘timely, relevant and structured clinical handover’ using standardised processes and information data sets to support safe patient care. However, this study found limitations in the comprehensiveness and quality of the information transferred. Limitations included poorly completed summaries and a format which prevented inclusion of additional individualised information, particularly about social and emotional wellbeing. In a state wide review of discharge summaries in Queensland [43] researchers found that while women’s basic personal information was included, many discharge summaries did not include information about support services women required or were accessing.

In our study, discharge summaries are most often completed by the midwife caring for woman on the day of discharge. Discharge summaries are sent from maternity within two days of discharge and received by the CFH nursing service within five days (and up to 10 days) of discharge. Information transfer for families with identified risk occurs via the same process, although additional phone contact is regularly made on behalf of these families in two states (VIC and TAS).

Barimani and Hylander [44] highlighted the importance of individualised communication transfer processes for children and families with identified risk. In phase one CFH nurses complained that ‘poor’ communication resulted in them being left unsure of the medical histories, existing plans and further management required for families with risk factors. This was confirmed in phase two with midwives and CFH nurses reporting that discharge summaries lacked important information on client social and emotional problems and other individualised client information. This was particularly problematic when women gave birth in the private sector. Psychosocial assessment was reported to be undertaken routinely by midwives employed in the public and private sectors (86.% public, 52.3% private) but was poorly reported in discharge summaries (38.9% public, 15.9% private). Similar findings were reported by Jenkinson and colleagues [43].

When information is not transferred women are required to either repeat their story or choose not to reveal important aspects of their history to the next health professional. This creates the potential for families with risk factors being left unsupported. Rollans et al. [45], identified that CFH nurses believed they were ‘*well equipped*’ when information on psychosocial needs was forwarded at ToC, while others confirmed that they were not always ‘*forewarned*’ and they ‘*don’t know what they will find*’ when information was not transferred [45].

Home-based postnatal care provided by midwives is reported by women as helpful and satisfying [46]. Most hospitals provide midwifery home visiting services to women within the first week of discharge. However, information collected at midwifery home visits was not routinely included on discharge summaries and midwives reported difficulties contacting CFH nurses to provide additional information. Difficulties with contacting CFH nurses may have been exacerbated by a higher percentage of CFH nurses in part-time employment (56.6% of respondents). Several studies have referred to staff shortages or inexperienced staff completing documentation also contributing to the poor quality of information transferred [13,47].

The review by Jenkinson et al [43] of the discharge process in Queensland also found that discharge summaries did not capture information on care provided by domiciliary services [48]. CFH nursing services first contact with families was usually made within the first two weeks (and up to 4 weeks) of discharge. Contact is not necessarily a home visit but may be a phone call used by the CFH nursing service to assess whether a home visit is required. Families instructed to contact CFH nursing services themselves may never connect. Therefore, families at risk and who might benefit most from a home visit and ongoing services are at additional risk because they are unlikely to contact services [49,50].

In this study, we found a level of tension exists between midwives and CFH nurses which impacted on the ToC. This tension has been reported elsewhere [51]. While this tension was most often linked to problems with the transfer of information, tension seemed to be influenced by the difference in each group’s philosophy of care, and a lack of understanding on both sides of the qualifications, role and contribution each brings to their work. Homer et al [12] also identified a lack of respect and understanding from midwives and CFH nurses of one another’s role and expertise. Professional role identity is essential for successful interprofessional working [52]. Interprofessional conflict may result without clarity around public professional identity [53]. Ineffective, information transfer therefore directly affect the success of ToC in a practical sense but may also affect the development of a trusting relationship between the client and health professional involved at that point.

Much of the tension between the two professional groups seems to be generated by the overlap of services during the postnatal period. Home visits by midwives occur in the first one or two weeks post discharge (up to six weeks in some services) and many CFH nursing services reported contacting women within 5 days of birth. Gaps existed in other areas where CFH nursing services do not receive sufficient information from maternity services to screen women for need via an initial visit or are unable to visit until 4 weeks. Similarly, in a qualitative study evaluating a quality improvement initiative to improve in-patient postnatal care and processes to transfer women home, midwives providing postnatal care at home reported their first home visit taking longer as a consequence of incomplete hospital discharge notes [27].

CFH nurses reported additional problems connecting with families when babies had been admitted to special care and neonatal intensive care nurseries and when women were discharged from private hospitals. Private hospitals were particularly problematic with limited or no psychosocial information, inefficient transfer of information, lack of referral to community services, and very few hospitals offering midwifery home visiting. Similar findings were noted by Jenkinson et al [43] who reported significant gaps in the content of discharge summaries, particularly in psychosocial and cultural information, and post-dsicharge advice [48].

Our understanding of the way in which the combination of neurological, biological and environmental factors influence development and long term outcomes for children has increased dramatically over the last decade [54,55]. Adverse events and/or chronic deprivation have been linked to poor behavioural outcomes, physical and mental ill health in adolescence and adulthood [56,57]. An individual’s response to stress is determined by both the cumulative effect and the level of stress exposure [58,59]. This realisation highlights the need to identify families with children at risk for adverse experience in early childhood. The concept of proportionate universalism whereby a basic level of health and educational supports in the early years are available to all families, with more intensive supports offered through negotiation with families when a need is identified is now being promoted as the most effective system-wide approach to meet the needs of all children and families [60,61].

However as phase one [32] and two of this study has demonstrated, delivery of a universal health service remains problematic due to poor communication systems, insufficient resources, the inadequate interface between private and public health systems and poor collaboration between professionals and services. Addressing these gaps in service continuity at ToC is therefore crucial. Ideally intervention should be initiated early, while brain plasticity is at its height making change possible [60]. Support and/or intervention should be provided during the antenatal and very early childhood period. Attempting to intervene at a later stage of development is more costly and less effective [60]. Ensuring women and families connect and remained connected to the maternity and CFH service is therefore important.

### Strategies to improve ToC

Hesselink, et al [62] undertook a systematic review of interventions to improve patient handovers from hospital to primary care. All but two studies included multicomponent interventions that used a comprehensive program, model or a liaison role. Specific strategies used within interventions included a liaison person, timely transfer of discharge summaries and client information, face-to-face or telephone case conferences [62]. Similar strategies were identified in our study, particularly for women and families with additional needs or risk factors for poor social and emotional health.

Strategies to improve the transition process should include changes in the information transfer process at both ends of the ToC process. Some of the tensions expressed by midwives regarding the CFH service appear to surround their inability to contact the CFH service when required. Likewise CFH nurses are unable to feedback to maternity services on subsequent care of families. The redesign of the client data collection tool to reflect individual needs of women is required [48]. Jenkinson et al [43] also advocate increased involvement of women in the completion of their own discharge summaries, providing women with the opportunity of discussing options for their post natal care.

As part of the National Maternity Service plan (2010) for the middle years 2012-2013, the Australian Health Ministers Advisory Council (AHMAC) made several commitments aimed at improving referral and information transfer from maternity services to CFH services. These included the introduction of the pregnancy hand-held record which is currently available in many Australian jurisdictions with attempts at a standardised national form currently underway [63]. These records, however, only record information provided before birth. To be useful in the postnatal period would require a redesign to include birth and postnatal details. The second AHMAC commitment was to map existing maternity service to CFH service information transfer and referral processes; however no significant progress has been made. Other varieties of health data linkage systems currently exist in several states (New South Wales, South Australia, Western Australia and Victoria) [64]. Generation of discharge summaries from women’s electronic record are recommended to facilitate the timely transfer of consistent and comprehensive information on both women and their babies to professionals providing post natal care [43]. A shared electronic record was been proposed nationally to rectify the communication and information transfer problems [65].

### Strengths and limitations

This study is the first large national study of transition of care from maternity to CFH services in Australia and as far as we are aware internationally. A mixed methods design enabled us to use a qualitative approach in phase one to identify issues from the perspective of midwives and CFH nurses. These issues were subsequently able to be confirmed and explored by a larger number of midwives and CFH nurses, using a quantitative approach in phase two.

This study is limited by several factors. This paper reports the perspectives of only two (midwives and CFH nurses) of the groups of professionals providing universal health services to women and families. Every effort was made to enrol similar numbers of midwives and CFH nurses from the various states and jurisdictions across Australia. Although participant numbers were large (655 midwives and 1098 CFH nurses) the response rate varied between states and jurisdictions. As participation in the study was voluntary, we also acknowledge that participants who agreed to be interviewed may have different views to midwives and CFH nurses who did not participate. Therefore participant responses may not be representative of all Australian midwives and CFH nurses and as such the study findings cannot be generalised broadly. However, the findings of phase two confirmed phase one findings and they are also consistent with the limited literature available regarding ToC across the maternity and child and family health continuum.

## Conclusion

Despite the existence of formal and informal models of transition there are significant problems with ToC for maternity services to CFH services in Australia. Improved ToC will require substantial changes in the information transfer process. A generic discharge summary designed to include psychosocial and biophysical information with the capacity to include additional information individualised to each women is necessary. A woman centred approach to discharge planning should be implemented. This would include encouraging the participation of women in planning their own postnatal care, the completion and dissemination of the discharge summary to services identified by the woman as providing ongoing support. In addition, families with identified risk factors require development of individualised processes for transition to CFH services initially and then onto additional services as required. Effective and more frequent communication between health professionals and the timely transfer of client information are essential to ensure continuity of care between midwifery services and other CFH professionals. Finally, the roles and responsibilities of health professionals in the provision of maternity and child and family health require clarification to avoid duplication of services and to lessen the current tension around role boundaries.

## Abbreviations

MCaFHNA: Australian Association of Maternal, 239 Child and Family Health Nurses; ACM: Australian College of Midwives; AHMAC: Australian Health Ministers Advisory Council; CFH nurse: Child and family health; CHoRUS: The Child Health: Researching Universal Service study; SCN: Special Care Nursery; ToC: Transition of care; UWS: University of Western Sydney.

## Competing interests

The authors declare that they have no competing interests.

## Authors’ contributions

KP: participated in the research design, data collection, data analysis and drafting of the manuscript completed the drafting of the paper, SK: participated in the study design and paper focus, helped draft the paper and provide critical review and guidance. CF: participated in the study design and paper focus, helped draft the paper and provide critical review and guidance. CH: participated in the study design and, and provided critical review and guidance on the paper. All authors read and approved the final manuscript. VS: conceived and participated in the study design and paper focus, helped draft the paper and provide critical review and guidance.

## Pre-publication history

The pre-publication history for this paper can be accessed here:

http://www.biomedcentral.com/1471-2393/14/151/prepub

## References

[B1] Reid R Haggerty J McKendry R Defusing the confusion: Concepts and measures of continuity of healthcare 2002 Montreal: Canadian Health Services Research Foundation

[B2] Australian Health Ministers Advisory Council National framework for universal child and family health services 2011 Canberra: Australian Government Retrieved from https://www.health.gov.au/internet/main/publishing.nsf/Content/nat-fram-ucfhs

[B3] Li Z Zeki R Hilder L Sullivan EA Australia’s mothers and babies 2010 2012 Canberra: AIHW

[B4] Henderson J Hornbuckle J Doherty D Models of Maternity Care: A Review of the Evidence. Women and Infants Research Foundation 2007 Western Australia: King Edward Memorial Hospital for Women

[B5] Schmied V Kruske S Barclay L Fowler C National Framework for Universal Child and Family Health Services (Final draft) Book National Framework for Universal Child and Family Health Services (Final draft) 2011 City: Australian Health Ministers’ Conference

[B6] NSW Department of Health NSW Heatlh/Families NSW:Supporting Families Early Package - SAFE START Guidelines: Improving Mental Health Outcomes for Parents and Infants 2009 Sydney: NSW Department of Health

[B7] COAG Investing in the Early Years—A National Early Childhood Development Strategy 2009 1 41

[B8] Brinkman SA Gialamas A Rahman A Mittinty MN Gregory TA Silburn S Goldfeld S Zubrick R Carr V Janus M Hertzman C Lynch JW Jurisdictional, socioeconomic and gender inequalities in child health and development: analysis of a national census of 5-year-olds in Australia BMJ Open 2012 2 e001075 22952161 10.1136/bmjopen-2012-001075 PMC3437432

[B9] The Nursing and Midwifery Office Health System Support Division Child and Family Health Nursing Professional practice framework 2011–2016 Book Child and Family Health Nursing Professional practice framework 2011–2016 2011 City: NSW Department of Health

[B10] Schmied V Donovan J Krusk S Kemp L Homer C Fowler C Commonalities and challenges: A review of Australian state and territory maternity and child health policies Contemp Nurse 2011 40 106 117 10.5172/conu.2011.40.1.106 22545909

[B11] Jeyendra A Rajadurai J Chanmugam J Trieu A Naiz S Baskaran R Schmied V Australian general practitioners’ perspectives on their role in well-child health care BMC Family Pract 2013 14 2 1 7 10.1186/1471-2296-14-2 PMC3542277 23282013

[B12] Homer C Henry K Schmied V Kemp L Leap N Briggs C 'It looks good on paper': Transitions of care between midwives and child and family health nurses in New South Wales Women Birth 2009 22 64 72 10.1016/j.wombi.2009.01.004 19217366

[B13] Barimani M Hylander I Linkage in the chain of care: a grounded theory of professional cooperation between antenatal care, postpartum care and child health care Int J Integr Care 2008 8 1 13 10.5334/ijic.254 PMC2638018 19209242

[B14] Axelsson R Axelsson SB Integration and collaboration in public health-a conceptual framework Int J Health Plann Manag 2006 21 75 88 10.1002/hpm.826 16604850

[B15] Cheng C Pickler R Effects of stress and social support on postpartum health of Chinese mothers in the United States Res Nurs Health 2009 32 582 591 10.1002/nur.20356 19877163

[B16] Bloom T Glass N Curry M Hernandez R Houck G Maternal stress exposures, reactions, and priorities for stress reduction among low-income, urban women J Midwifery Womens Health 2012 58 167 174 23278984 10.1111/j.1542-2011.2012.00197.x PMC4317357

[B17] Miller Y Thompson R Porter J Prosser S Findings from the Having a baby in Queensland Survey 2011

[B18] Fenwick J Gamble J Creedy D Barclay L Buist A Ryding EL Women's perceptions of emotional support following childbirth: A qualitative investigation Midwifery 2013 29 217 224 10.1016/j.midw.2011.12.008 23149239

[B19] Gaillard A Le Strat Y Mandelbrot L Keita H Dubertret C Predictors of postpartum depression:Prospective study of 264 women followed during pregnancy and postpartum Psychiatry Res 2014 215 341 346 10.1016/j.psychres.2013.10.003 24370337

[B20] Milgrom J Gemmill A Bilszta J Hayes B Barnett B Brooks J Ericksen J Ellwood D Buist A Antenatal risk factors for postnatal depression: A large prospective study J Affect Disord 2008 108 147 157 10.1016/j.jad.2007.10.014 18067974

[B21] Letourneau N Dennis C Benzies K Duffett-Leger L Stewart M Tryphonopoulos P Este D Watson W postpartum depression is a family affair: addressing the impact on mothers, fathers, and children Issues Ment Health Nurs 2012 33 445 457 10.3109/01612840.2012.673054 22757597

[B22] Beck CT The effects of Postpartum Depression on Maternal-infant interaction: a meta-analysis Nurs Res 1995 44 298 304 7567486

[B23] Leahy-Warren P McCarthy G Corcoran P Postnatal depression in first-time mothers: prevalence and relationships between functional and structural social support at 6 and 12 weeks postpartum Arch Psychiatr Nurs 2011 25 174 184 10.1016/j.apnu.2010.08.005 21621731

[B24] Manuel J Martinson M Bledsoe-Mansori S Bellamy J The influence of stress and social support on depressive symptoms in mothers with young children Soc Sci Med 2012 75 2013 2020 10.1016/j.socscimed.2012.07.034 22910191

[B25] MacArthur C Winter H Bick D Knowles H Lilford R Henderson C Lancashire R Braunholtz D Gee H Effects of redesigned community postnatal care on womens’ health 4 months after birth: a cluster randomised controlled trial Lancet 2002 359 378 385 10.1016/S0140-6736(02)07596-7 11844507

[B26] Henderson J Redshaw M Who Is Well After Childbirth? Factors Related to Positive Outcome BIRTH Issues Perinatal Care 2013 40 1 1 9 10.1111/birt.12022 24635418

[B27] Bick DE Rose V Weavers A Wray J Beake S Improving inpatient postnatal services: midwives views and perspectives of engagement in a quality improvement initiative Health Serv Res 2011 11 293 1 10 10.1186/1472-6963-11-293 PMC3215185 22044744

[B28] Eronen R Caabretto H Pincombe J Improving the professional support for parents of young infants Aust J Prim Health 2011 17 186 194 10.1071/PY10062 21645476

[B29] Boe Danbjørg D Wagner L Clemensen J Do families after early postnata ldischarge need new ways to communicate with the hospital?A feasibilility study Midwifery 2013 17 1 8 10.1016/j.midw.2013.06.006 23871291

[B30] Ludina SM Arbonb P Parkerb S Patients’ transition in the Intensive Care Units: Concept analysis Intensive Crit Care Nurs 2013 29 187 192 10.1016/j.iccn.2013.02.001 23727138

[B31] Thomas MJW Schultz TJ Hannaford N Runciman WB Failures in transition: learning from incidents relating to clinical handover in acute care J Healthc Qual 2013 35 40 56 10.1111/j.1945-1474.2011.00189.x 22268639

[B32] Psaila K Schmied V Fowler F Kruske S Discontinuities between maternity and child and family health services: health professional’s perceptions BMC Health Serv Res 2013 14 4 1 12 10.1186/1472-6963-14-4 PMC3893500 24387686

[B33] Cowley S Caan W Dowling S Weir H What do health visitors do? a national survey of activities and service Publlic Health 2007 In Press 10.1016/j.puhe.2007.03.016 17606280

[B34] The National Transitions of Care Coalition Measures Work Group Transitions of Care Measures 2008 http://www.ntocc.org/WhoWeServe/HealthCareProfessionals.aspx

[B35] Haggerty J Roberge D Freeman G Beaulieu C Experienced continuity of care when patients see multiple clinicians: a qualitative metasummary Ann Fam Med 2013 11 262 271 10.1370/afm.1499 23690327 PMC3659144

[B36] Haggerty J Roberge D Freeman G Beaulieu C Breton M Validation of a generic measure of continuity of care: when patients encounter several clinicians Ann Fam Med 2012 10 443 451 10.1370/afm.1378 22966108 PMC3438212

[B37] Graneheim VH Lundman B Qualitative content analysis in nursing research: concepts, procedures and measures to achieve trustworthiness Nurse Educ Today 2004 24 105 112 10.1016/j.nedt.2003.10.001 14769454

[B38] NSW Department of Health Book NSW Supporting Families Early Package - SAFE START Strategic Policy 2009 City: NSW Department of Health

[B39] Eastwood J Kemp L Jalaludin B Explaining ecological clusters of maternal depression in South Western Sydney BMC Pregnancy Childbirth 2014 14 47 1 15 24460690 10.1186/1471-2393-14-47 PMC3909479

[B40] Australian College of Midwives Transition to maternal and child health services 2007, 3rd June, 2013 Retrieved 20th May, 2013, from http://www.midwives.org.au/lib/pdf/documents/Transition%20to%20Maternal%20and%20Child%20Health%20Services.pdf

[B41] Victorian Health Department Postnatal Care Program Guidelines for Victorian Health Services 2012 Melbourne: The Performance, Acute Programs and Rural Health branch, Victorian Government

[B42] ACSQHC National Safety and Qualtiy Health Service Standards 2012 Sydney: Australian Commission on Safety and Quality in Health Care

[B43] Jenkinson B Young K Kruske S Maternity services and the discharge process: a review of practice in Queensland, Australia Women Birth 2013 in press 10.1016/j.wombi.2013.12.001 24373729

[B44] Barimani M Hylander I Joint action between child health care nurses and midwives leads to continuity of care for expectant and new mothers Int J Qualitative Stud Health Well Being 2012 18183 1 11 10.3402/qhw.v7i0.18183 PMC3391663 22783367

[B45] Rollans M Schmied V Kemp L Meade T Negotiating policy in practice: child and family health nurses’ approach to the process of postnatal psychosocial assessment BMC Health Serv Res 2013 13 7 10.1186/1472-6963-13-7 23565716 PMC3637412

[B46] Fenwick J Butt J Satvinder D Hauck Y Schmied V Western Australian women’s perceptions of the style and quality of midwifery postnatal care in hospital and at home Women Birth 2010 23 10 21 10.1016/j.wombi.2009.06.001 19632912

[B47] Beake S Rose V Bick D Weavers A Wray J A qualitative study of the experiences and expectations of women receiving in-patient postnatal care in one English maternity unit BMC Pregnancy Childbirth 2010 10 70 1 9 20979605 10.1186/1471-2393-10-70 PMC2978124

[B48] Mickan S Hoffman SJ Nasmith L Collaborative practice in a global health context: Common themes from developed and developing countries J Interprof Care 2010 24 492 502 10.3109/13561821003676325 20718595

[B49] Ammerman R Stevens J Putnam F Altaye M Hulsmann J Lehmkuhl H Monroe J Gannon T Ginkel J Predictors of Early engagement in Home visitation J Fam Violence 2006 21 105 115 10.1007/s10896-005-9009-8

[B50] Wen LM Orr N Rissel C The role of ethnicity in determining access to and acceptability of home visiting for early childhood health and wellbeing Aust Health Rev 2007 31 132 139 10.1071/AH070132 17266497

[B51] Colvin C de Heer J Winterton L Mellenkamp M Glenton C Noyes J Lewin S Rashidian A A systematic review o fqualitativeevidence on barriers and facilitators to the implementation of task-shifting in midwifery services Midwifery 2013 20 1211 1221 23769757 10.1016/j.midw.2013.05.001

[B52] Hind M Cooper I Gill S Hilton R Judd P Jones N Interprofessional perceptions of health care students J Interporfessional Care 2003 17 21 34 10.1080/1356182021000044120 12772467

[B53] Machin A Machin T Pearson P Maintaining equilibrium in professional role identity: a grounded theory study of health visitors’ perceptions of their changing professional practice context J Adv Nurs 2011 68 1526 1537 22211526 10.1111/j.1365-2648.2011.05910.x

[B54] Nelson HJ Kendal G Shields L Neurological and biological foundations of children’s social and emotional development: an integrated literature review J Sch Nurs 2013 201 1 10 10.1177/1059840513513157 24257899

[B55] Shonkoff JP Leveraging the biology of adversity to address the roots of disparities in health and development Proc Nat Acad Sci U S A 2012 109 2 17103 17107 10.1073/pnas.1121259109 PMC3477384 23045654

[B56] Flaherty E Thompson R Litrownick A Theodore A English D MM B Wike T Whimper L Runyan D Dubowitz H Effect of early childhood adversity on child health Arch Pediatr Adolesc Med 2006 160 1232 1238 10.1001/archpedi.160.12.1232 17146020

[B57] Shonkoff J Garner A The lifelong effects of early childhood adversity and toxic stress Pediatrics 2012 129 1 e232 e246 10.1542/peds.2011-2663 22201156

[B58] Garner A Home visiting and the Biology of Toxic Stress: opportunities to Address Early Chilhood Adversity Pediatrics 2013 132 Suppl 2 S65 73 24187125 10.1542/peds.2013-1021D

[B59] Shonkoff J Thomas Boyce W McEwen BS Neuroscience, Molecular Biology, and the Childhood Roots of Health Disparities Building a New Framework for Health Promotion and Disease Prevention JAMA 2009 301 21 2252 2259 10.1001/jama.2009.754 19491187

[B60] Oberklaid F Baird G Blair M Melhuish E Hall D Children's health and development: approaches to early identification and intervention Arch Dis Child 2013 98 12 1008 1011 10.1136/archdischild-2013-304091 23968776

[B61] Marmot M Atkinson T Bell J Black C Broadfoot P Cumberlege J Mulgan G Fair Society, Healthy Lives: A Strategic Review of Health Inequalities in England Post-2010 2010 London: The Department of Health Retrieved from "http://www.ucl.ac.uk/marmotreview"

[B62] Hesselink G Schoonhoven L Barach P Spijker A Gademan P Kalkman C Liefers J Vernooij-Dassen M Wollersheim H Improving patient handovers from hospital to primary care: a systematic review Ann Intern Med 2012 157 417 428 10.7326/0003-4819-157-6-201209180-00006 22986379

[B63] World Health Organization Framework for Action on Interprofessional Education and Collaborative Practice 2010 Geneva, Switzerland 21174039

[B64] Walker J Welfare AIH Maternity data in Australia: a review of sources and gapsvol. 87 2011 Canberra: AIHW National Perinatal Epidemiology and Statistics Uni

[B65] Commonwealth Government National Maternity Services Plan 2011 Canberra: Austraila Health Ministers conference 1 127

